# Geometric semi-automatic analysis of radiographs of Colles’ fractures

**DOI:** 10.1371/journal.pone.0238926

**Published:** 2020-09-14

**Authors:** Constantino Carlos Reyes-Aldasoro, Kwun Ho Ngan, Ananda Ananda, Artur d’Avila Garcez, Andrew Appelboam, Karen M. Knapp

**Affiliations:** 1 School of Mathematics, Computer Science and Engineering, City, University of London, London, United Kingdom; 2 Royal Devon and Exeter Hospital, Exeter, United Kingdom; 3 College of Medicine and Health, University of Exeter, Exeter, United Kingdom; University Kuala Lumpur, MALAYSIA

## Abstract

Fractures of the wrist are common in Emergency Departments, where some patients are treated with a procedure called Manipulation under Anaesthesia. In some cases, this procedure is unsuccessful and patients need to revisit the hospital where they undergo surgery to treat the fracture. This work describes a geometric semi-automatic image analysis algorithm to analyse and compare the x-rays of healthy controls and patients with dorsally displaced wrist fractures (Colles’ fractures) who were treated with Manipulation under Anaesthesia. A series of 161 posterior-anterior radiographs from healthy controls and patients with Colles’ fractures were acquired and analysed. The patients’ group was further subdivided according to the outcome of the procedure (successful/unsuccessful) and pre- or post-intervention creating five groups in total (healthy, pre-successful, pre-unsuccessful, post-successful, post-unsuccessful). The semi-automatic analysis consisted of manual location of three landmarks (finger, lunate and radial styloid) and automatic processing to generate 32 geometric and texture measurements, which may be related to conditions such as osteoporosis and swelling of the wrist. Statistical differences were found between patients and controls, as well as between pre- and post-intervention, but not between the procedures. The most distinct measurements were those of texture. Although the study includes a relatively low number of cases and measurements, the statistical differences are encouraging.

## Introduction

The dorsally displaced wrist fracture, also known as Colles’ fracture, is the most common fracture involving a metaphyseal fracture leading to a posterior displacement of the distal fragment [1, 2]. This can result in some residual impairment in the motion of the hand and wrist [3], and more serious complications such as neuropathies, arthrosis, tendon ruptures and finger stiffness [4].

The main procedures for these fractures are Manipulation under Anaesthesia (MUA) and open surgery, also known as Open Reduction and Internal Fixation (ORIF) [5]. MUA, which includes closed reduction and casting, [5], is often the primary option undertaken in Emergency Departments for the displaced fractures in an attempt to correct the deformity and represents a significant proportion of the department workload [6]. Patients are initially treated with a temporary plaster cast after manipulation and a follow-up visit to monitor the rehabilitation progress on a separate day. In general, the fractured position would be improved upon manipulation. There are however cases where the fracture remains unstable or, despite plaster cast immobilisation, slip back into an unacceptable position during rehabilitation. ORIF would then have to be performed with yet another hospital visit causing significant inconvenience to the patient and further inefficiency to hospital resources. Nevertheless, ORIF is generally, not the preferred option as it requires the booking of operating theatre to operate on the manipulation and fixation of metallic pins, plates or screws. The ORIF procedure is also more complicated than MUA, and can lead to serious complications [7].

Clinicians determine the need to manipulate wrist fractures upon clinical and radiographical grounds. New, visible wrist deformity is a widely accepted clinical indication for manipulation. Radiographic assessment is routinely performed through evaluation of radiographs with lateral and postero-anterior (PA) views, which are used to detect, classify, assess fracture severity and evaluate treatment options [8–10]

When assessing radiographs of fractures, a series of geometric measurements have been proposed, namely, volar tilt, radial inclination, ulnar variance, [11], radial length [12], radial height, ulnar variance, articular stepoff [13] and metaphyseal collapse ratio [14]. These measurements have been used to assess the clinical outcome of elderly patients [15, 16]. Non-geometric measurements such as bone density have also been used to assess the fragility of bones [17, 18].

Although there are quoted radiographic deformities suggesting the need for wrist fracture reduction including dorsal tilt, dorsal displacement, loss of radial height and radial deviation from the anatomical position, in practice, these are not formally measured. Rather, many clinicians will take into account the position of the fracture on x-ray as a whole and the functional status of the patient including the current and future demands of wrist use when deciding on whether to manipulate the fracture, in consultation with the patient.

Despite considerable research [5–7, 19–22], there is still ambiguity in the procedure to follow with Colles’ fracture [23–25]. There is some evidence that the degree of initial deformity and other factors such as age, dependency, functional status and presence of osteoporosis and x-ray characteristics like axial shortening of bones [26, 27] and angles of volar tilt [28] might predict instability [29–33].

It is common that the radiographic measurements previously mentioned are obtained manually by radiologists or radiographers. Whilst these measurements are still considered as gold standard, the manual delineation of measurements is laborious and subject to inter- and intra-observer variability and some times measurements are repeated by more than one person to obtain more reliable measurements [34–36]. This work describes a geometric semi-automatic image analysis algorithm to analyse and compare the radiographs of healthy controls and patients with Colles’ fractures who have undergone either MUA and were followed to determine if the procedure was successful or unsuccessful. The measurements are obtained in a semi-automatic way in which three landmarks are selected by a user in a matter of seconds, and then the measurements are generated automatically with image-processing algorithms. The process is much faster than obtaining all measurements manually.

The main objective of this work is to determine if there are geometric differences between the successful and unsuccessful cases. The semi-automatic comparisons extracted a series of measurements, e.g. widths of forearm and metacarpal, based on three manually-placed landmarks. In particular, texture measurements at the radial bone were also explored. More accurate computer assisted assessment of fracture position together with evidence based decision making algorithms have the potential to save health services time and money whilst ensuring the best outcomes for patients.

## Materials and methods

### Study design and patients

In this work, one hundred and sixty-one posterior-anterior radiographs of wrist fractures from previous internal department activity were analysed. The study was submitted to the Health Research Authority (HRA) of National Health Service of the United Kingdom for approval through the Integrated Research Application System (IRAS). The study was approved with the IRAS Project ID: 233195. The data corresponding to the radiographs that were analysed was anonymised following the ethics procedures at the donating institution. It was sourced ethically, with Caldicott Guardian approval, from the Royal Devon and Exeter Hospital. All MUAs were undertaken or supervised by fully accredited emergency clinicians (consultants).

**Fig 1:**
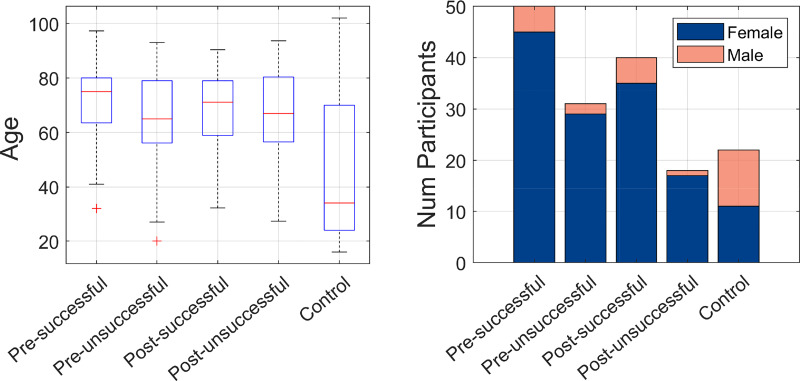
Graphical illustration of the demographic distribution of the population of the study. (a) Age distribution is shown with boxplots, one per group of the study. (b) Female and Male distribution is shown with bars, one per group of the study.

Of the 161 radiographs, 139 corresponded to wrist fractures and 22 to healthy controls. The controls corresponded to patients who required wrist radiographs, mostly following injury, to rule out fractures. As these cases did not present fractures, they were considered as healthy and used as controls. The wrist fractures were divided by the acquisition time: before (Pre) or after (Post) MUA and the outcome of these: successful or unsuccessful therefore creating four classes pre-successful (n = 50), pre-unsuccessful (n = 31), post-successful (n = 40), post-unsuccessful (n = 18). These cases and the clinical outcome were retrospectively identified from electronic attendance logs and electronic records. The demographics of the participants in the study are illustrated in Fig 1.

### X-ray acquisition

X-rays were obtained with five different x-ray units: DigitalDiagnost DidiEleva01 (Philips Medical Systems, Netherlands), Mobile tablet work station (Thales, France), DirectView CR 975 and CD 850A (Kodak, USA), Definium 5000 (GE Healthcare, USA) with a variety of exposure factors and saved in DICOM format [37].

**Fig 2:**
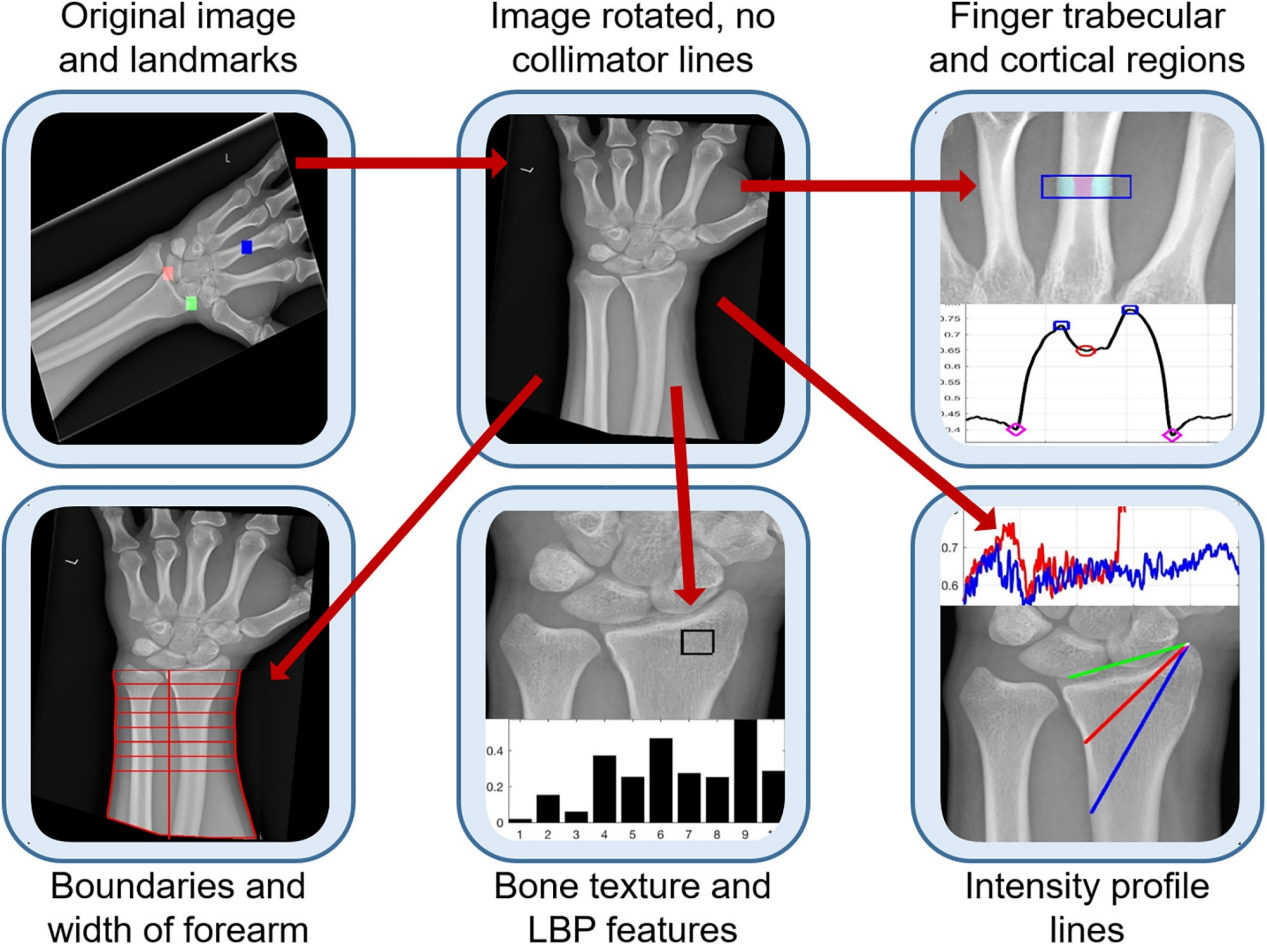
Six representative radiographs that were collected from previous clinical activity at Royal Devon and Exeter NHS foundation trust emergency department. The images present considerable variability in the quality, positioning of the arm and presence of lines caused by the x-ray collimator. The images were anonymised and metadata such as age, date of acquisition, gender and clinical outcome was available.

Six representative cases of the radiographs are shown in Fig 2. The radiographs presented considerable variability in the quality, positioning of the arm and presence of collimation lines.

### Image analysis

**Fig 3:**
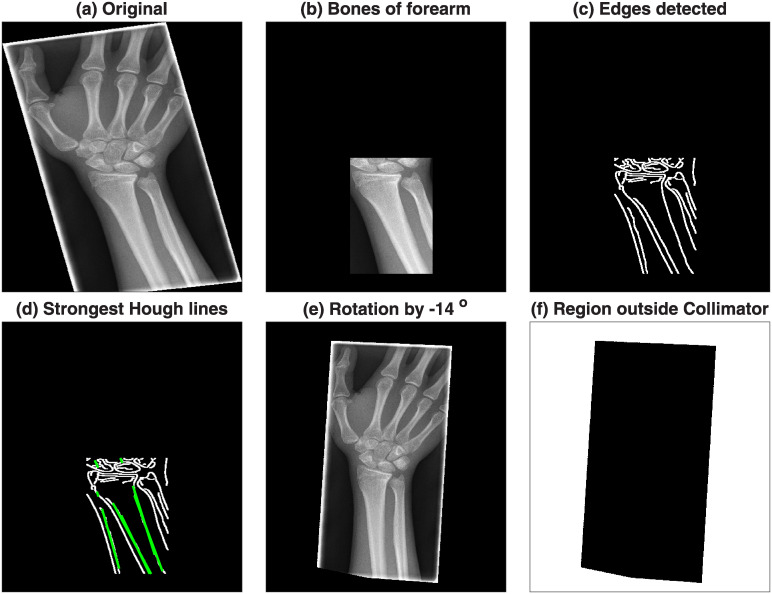
Graphical illustration of the steps for image analysis. Radiographs are first pre-processed by manually selecting three landmarks and then automatically rotating so that the bones of the forearm are vertical and the lines produced by the collimator are removed. Then, measurements are extracted from four regions: the bone of the middle finger, the edges of the forearm, a textured region of the radius and two intensity profile lines over the radius.

The analysis is considered semi-automatic as three landmarks are manually located, and the algorithms obtain all the measurements. A graphical illustration the image analysis steps is presented in Fig 3. All the code was developed in Matlab^®^ (The MathworksTM, Natick, MA, USA) and is available open-source in GitHub (https://github.com/reyesaldasoro/fractures/).

**Fig 4:**
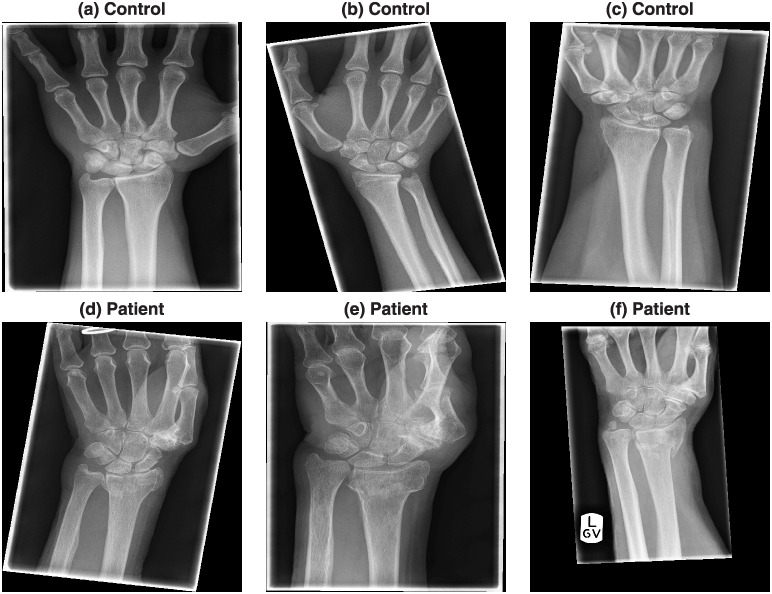
Pre-processing steps. (a) Original image, notice the lines of the collimator and the rotation of the radiograph. (b) Region of the bones of the forearm. (c) Edges of regions where regions show high change of intensity, detected with Canny algorithm. (d) Strongest lines detected by Hough transform overlaid on the edges. (e) Original image rotated by the angle of the lines detected in (d). (f) Region outside the collimator labelled in white. This region will be dilated to remove the lines of the collimator.

**Fig 5:**
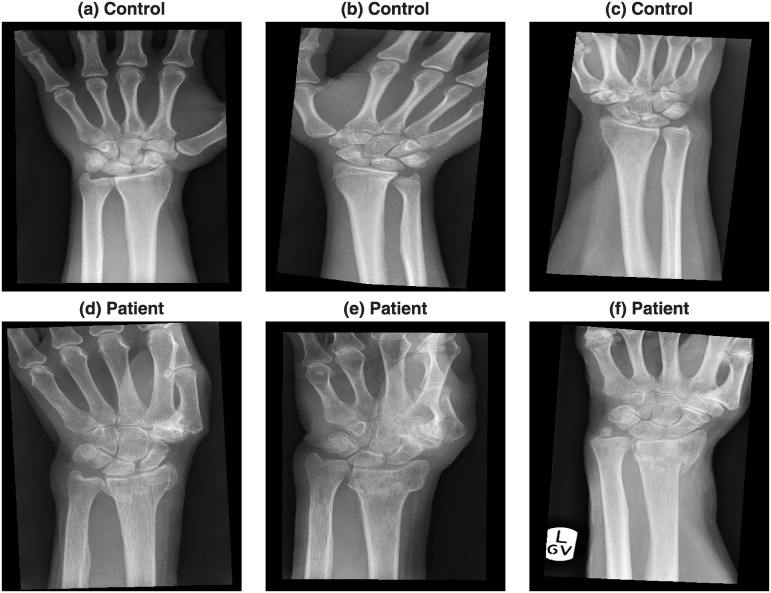
Outcome of the automatic pre-processing of the radiographs. The six representative cases shown in Fig 2 were automatically rotated so that the forearm is vertical. In addition, the artefacts due to the collimator were removed. All the code is written in Matlab and each image is pre-processed in less than one second.

The pre-processing step removed the lines caused by the collimator and then aligned the forearm vertically. For this, the DICOM images and headers were read, converted into Matlab and saved as a *.mat file. The central region of the radiograph was selected by dividing the rows and columns into three thirds and selecting the central part where the bones of the forearm were prominent (Fig 4a and 4b). Lines of regions where there is abrupt change of intensity were detected through the Canny edge detection algorithm [38] (Fig 4c). was applied and thus The strongest lines (i.e. those that longest and most straight so that they will provide the highest projection when summed over a profile line) of the bones of the forearm were considered a good indication of the orientation of the arm. These strongest lines were detected with the use of the Hough transform [39]. The Hough transform can be understood as a technique in which a profile line is sequentially rotated around a pivot point and then it is displaced and rotated again and again. Eventually, the profile lines will cover the whole image and the sum of the intensity of all profiles is used to calculate where lines are located over the image. The strongest lines are overlaid on the edges in Fig 4d. The median angle of these lines was used to determine the rotation required to align the forearm vertically (Fig 4e). The lines of the collimator were easily detected as the pixels that were beyond the lines of the collimator were always zero, whilst the darkest regions inside the lines of the collimator, whilst low, were always above zero. Thus, the region(s) outside the lines were detected, dilated and removed from the image (Fig 4f). Fig 5 shows the effect of the pre-processing in the six cases of Fig 2.

**Fig 6:**
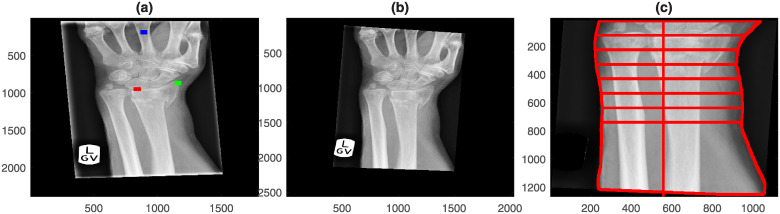
Semi-automatic extraction of measurements of the forearm. (a) Original radiograph that presents rotation of the arm and artefactual lines due to the collimator. Three landmarks have been manually located in the base of the lunate (red), radial styloid (green) and centre of the middle finger (blue). (b) Automatic pre-processing of the image where the forearm was aligned vertically and the lines removed. (c) Using the lunate landmark as a guide, the boundaries of the forearm were automatically delineated and lines traced between the boundaries. The distance between the lines is 1 cm and were being used to derive swelling measurements of the wrist.

Every image was displayed and three landmarks were manually selected in the following order: (1) base of the lunate, (2) extreme of the radial styloid, (3) centre of the metacarpal of the middle finger (Fig 6a). These landmarks were subsequently used to obtain a series of measurements described below.

Three groups of measurements were analysed with the expectation that each of these groups would correlate with a clinical condition such as swelling or osteoporosis.

First, as an indication of swelling, the boundaries of the forearm were detected. The landmark of the lunate (Red dot in Fig 6a) was used to determine the base of the wrist. The region of interest was determined from this point towards the forearm, and the region of the hand was removed. The boundaries of the forearm were detected by Canny edge detection, and then 8 lines perpendicular to the forearm, each at 1 cm separation were traced. The width of the forearm at each of these lines was recorded (Fig 6c) with the conjecture that the relationship between the widths could be an indication of swelling of the wrist due to the fracture. A series of ten measurements were generated by calculating ratios, e.g. width at the centre divided by widths at the extremes.

**Fig 7:**
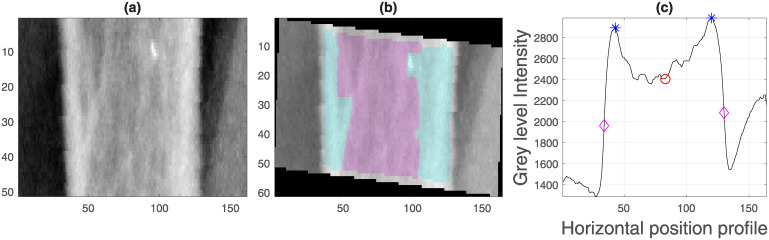
Semi-automatic extraction of measurements of the finger (a) Region of interest (ROI) of the central finger generated from the landmark, blue dot in Fig 5a. (b) Identification of regions of cortical bone (shaded in cyan) and trabecular bone (shaded in pink) from which the ratio of cortical to total area was calculated. Notice that the finger was rotated to align vertically as the previous rotation aligned the forearm but the fingers are not necessarily vertical. (c) Intensity profile of the ROI with the following key points: edges of the bone (magenta diamond), peak of cortical bone (blue asterisk) and centre of bone (red circle).

Second, the landmark of the middle finger (blue dot in Fig 6a) was used to extract a region of interest that contained a segment of the finger (Fig 7a). The bone in this region was also aligned vertically. Then, the edges of the finger itself and the trabecular and cortical regions were obtained (Fig 7b) by calculating a vertical projection of the intensities of the image (Fig 7c). It was conjectured that the thickness of the cortical and trabecular regions of the bone would be an indication of osteoporosis [40–42]. The measurements extracted were the width of the finger and the ratio of trabecular area to total area.

**Fig 8:**
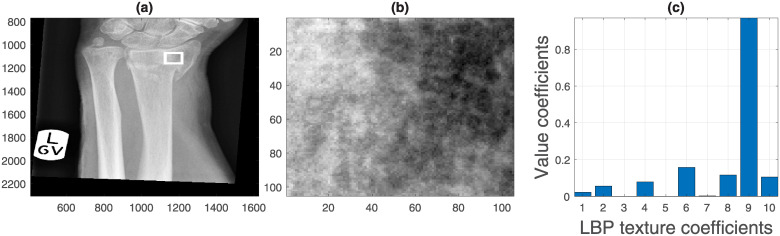
Semi-automatic extraction of texture measurements of a region of interest. (a) To analyse the texture of the radius, a ROI is automatically located by traversing a fixed distance from the radial styloid landmark. (b) Zoom of the region of interest. (c) Texture coefficients generated by Local Binary Pattern analysis.

Third, preliminary work had identified the potential correlation of texture measurements extracted from x-rays with image analysis with clinical outcome [43]. Therefore, bone texture was analysed in two ways. First, a small region of bone (Fig 8a and 8b) was selected from the radius, a short distance away from the landmarks previously detected. This region was analysed with a texture technique called Local Binary Pattern (LBPs) [44], which explores the relations between neighbouring pixels. LBPs concentrate on the relative intensity relations between the pixels in a small neighbourhood and not in their absolute intensity values or the spatial relationship of the whole data. The texture analysis was based on the relationship between the pixels of a 3 × 3 neighbourhood. A Texture Unit was calculated by differentiating the grey level of a central pixel with the grey level of its neighbours. The differences were measured if the neighbour is greater or lower than the central pixel and is then recorded as a histogram (Fig 8c). This analysis provided 10 measurements.

**Fig 9:**
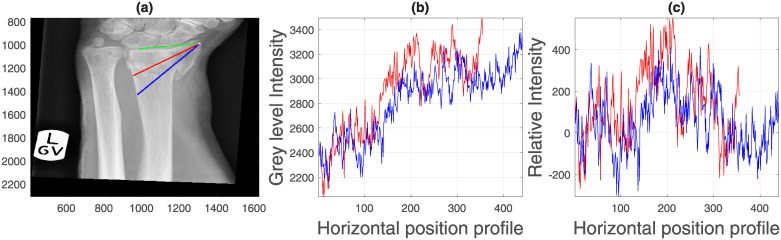
Semi-automatic extraction of texture measurements from intensity profiles. (a) Profile lines from the radial styloid. Initially, a line (green) is automatically traced between the lunate (red in Fig 5a) the radial styloid (green in Fig 5a) landmarks. Two lines are automatically derived from the first, one at 30 degrees (red) and one at 45 degrees (blue) from the radial styloid up to the edge of the radius, which is automatically detected. (b) Intensity profiles corresponding to the lines traced in (a). Notice the increasing slope. (c) Intensity profiles adjusted by removing the slope.

Another way of analysing the texture of the bones is through intensity profile lines, which capture the variation of the bone intensity over a straight line (Fig 9a). Initially, a line (green) was automatically traced between the lunate the radial styloid landmarks. Two lines were automatically derived from the first, one at 30 (red) and one at 45 (blue) degrees from the radial styloid up to the edge of the radius. The edge was automatically detected when the intensity dropped drastically into the darker region between the bones. Measurements were extracted both from the intensity profiles (Fig 9b) and also the profiles after these were adjusted by removing the slope (Fig 9c) with the idea that measurements like the standard deviation would not be biased by a line that increases its intensity. This analysis provided 10 measurements, e.g. length, slope and standard deviation of the profile.

All steps except the location of the three landmarks is automatic and takes around 10-20 seconds to process with custom-made Matlab scripts.

## Results

**Fig 10:**
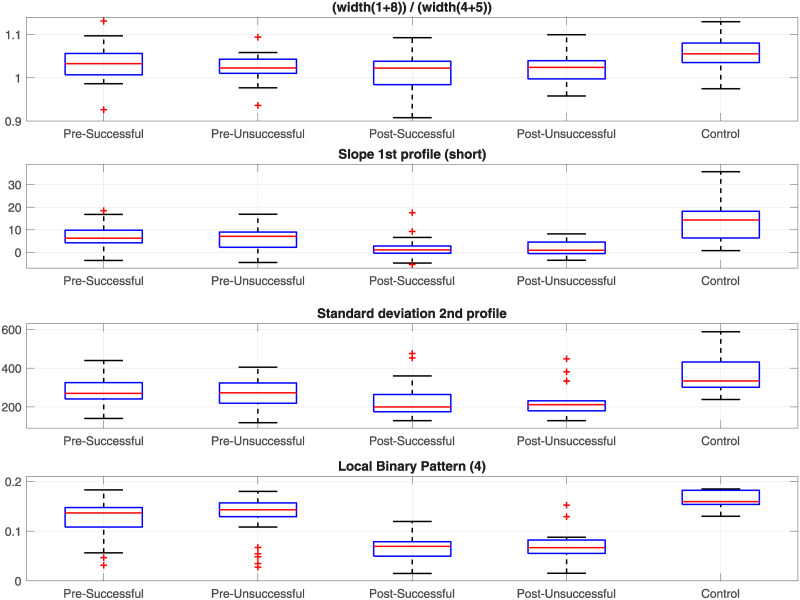
Boxplots corresponding to distributions of four representative measurements. The differences between control and patients and the pre- and post-intervention cases are noticeable but within the pre-intervention and post-intervention groups are very close to each other.

A total of 32 measurements were extracted for each of the radiographs of the five groups previously described, and these are presented in Table 1. For each of the measurements, statistical difference between the following cases was tested with paired t-tests: (i) healthy controls against patients. (ii) Pre-intervention (successful and unsuccessful) against post-intervention (successful and unsuccessful). (iii) Successful against unsuccessful. (iv) Pre-intervention successful against pre-intervention unsuccessful. (v) Post-intervention successful against post-intervention unsuccessful. (vi) Pre-intervention successful against post-intervention successful. (vii) Pre-intervention unsuccessful against post-intervention unsuccessful. Four representative measurements are shown in Fig 10 as boxplots.

**Table 1:** Measurements extracted from the radiographs. Third column corresponds to the landmark used to calculate the measurement. Columns 4-10 show the p-values result of paired t-tests between different groups. Values lower than 0.05 are highlighted in bold. Abbreviations: Ratio of width line 1 / width line 4 (W1 / W4), Local Binary Pattern (LBP), Standard Deviation (Std).

	Measurement	Landmark	Control v Patient	Pre- v Post-	Successful v Unsuccessful	Pre-Successful v Pre-Unsuccessful	Post-Successful v Post-Unsuccessful	Pre-Successful v Post-Successful	Pre-Unsuccessful v Post-Unsuccessful
1	W1 / W4	Lunate	< **0.01**	**0.01**	0.55	0.16	0.51	**0.01**	0.74
2	W2 / W4	Lunate	< **0.01**	0.21	0.96	0.53	0.44	0.09	0.99
3	W3 / W4	Lunate	**0.04**	0.36	0.37	0.37	0.68	0.19	0.78
4	W5 / W4	Lunate	0.10	0.30	0.38	0.19	0.33	0.94	0.09
5	W6 / W4	Lunate	0.22	0.37	0.39	0.30	0.76	0.88	0.27
6	W7 / W4	Lunate	0.92	0.31	0.41	0.53	0.68	0.49	0.51
7	W8 / W4	Lunate	0.45	0.25	0.65	0.50	0.77	0.54	0.33
8	Min width / Max width	Lunate	0.22	< **0.01**	0.16	0.39	0.41	< **0.01**	0.11
9	W1+W8 / W4+W5	Lunate	< **0.01**	**0.01**	0.72	0.21	0.72	< **0.01**	0.53
10	W1+W2 / W7+W8	Lunate	0.06	0.50	0.58	0.35	0.60	0.23	0.73
11	Trabecular Area / Total Area	Finger	< **0.01**	0.07	0.13	0.11	0.49	0.07	0.43
12	Width Finger	Finger	0.85	**0.02**	0.66	0.86	0.52	**0.04**	0.40
13	LBP 1	L+Rad Sty	**0.02**	**0.01**	0.28	0.96	0.07	**0.01**	0.54
14	LBP 2	L+Rad Sty	< **0.01**	< **0.01**	0.41	0.83	0.09	< **0.01**	**0.01**
15	LBP 3	L+Rad Sty	< **0.01**	< **0.01**	0.22	0.68	0.13	< **0.01**	**0.03**
16	LBP 4	L+Rad Sty	< **0.01**	< **0.01**	0.14	0.45	0.26	< **0.01**	< **0.01**
17	LBP 5	L+Rad Sty	< **0.01**	< **0.01**	0.16	0.46	0.13	< **0.01**	< **0.01**
18	LBP 6	L+Rad Sty	< **0.01**	< **0.01**	0.08	0.24	0.30	< **0.01**	< **0.01**
19	LBP 7	L+Rad Sty	< **0.01**	< **0.01**	0.17	0.60	0.07	< **0.01**	< **0.01**
20	LBP 8	L+Rad Sty	< **0.01**	< **0.01**	0.06	0.22	0.23	< **0.01**	< **0.01**
21	LBP 9	L+Rad Sty	< **0.01**	< **0.01**	0.15	0.57	0.11	< **0.01**	< **0.01**
22	LBP 10	L+Rad Sty	< **0.01**	< **0.01**	0.46	0.78	0.09	< **0.01**	**0.01**
23	Slope profile 1 (full line)	Radial Styloid	< **0.01**	< **0.01**	0.88	0.29	0.35	< **0.01**	0.09
24	Slope profile 2 (full line)	Radial Styloid	0.04	< **0.01**	0.39	0.78	0.52	< **0.01**	**0.02**
25	Slope profile 1 (short segment)	Radial Styloid	< **0.01**	< **0.01**	0.91	0.53	0.85	< **0.01**	< **0.01**
26	Slope profile 2 (short segment)	Radial Styloid	< **0.01**	0.06	0.82	0.74	0.85	0.11	0.29
27	Std profile 1	Radial Styloid	< **0.01**	< **0.01**	0.92	0.39	0.59	< **0.01**	**0.05**
28	Std profile 2	Radial Styloid	< **0.01**	< **0.01**	0.74	0.84	0.77	< **0.01**	0.06
29	Std profile 1 adjusted	Radial Styloid	< **0.01**	< **0.01**	0.50	0.80	0.47	< **0.01**	< **0.01**
30	Std profile 2 adjusted	Radial Styloid	< **0.01**	< **0.01**	0.10	0.42	0.22	< **0.01**	< **0.01**
31	Distance profile 1	Radial Styloid	< **0.01**	0.23	0.78	0.99	0.75	0.28	0.63
32	Distance profile 2	Radial Styloid	< **0.01**	0.25	1.00	0.98	0.86	0.42	0.38

**Fig 11:**
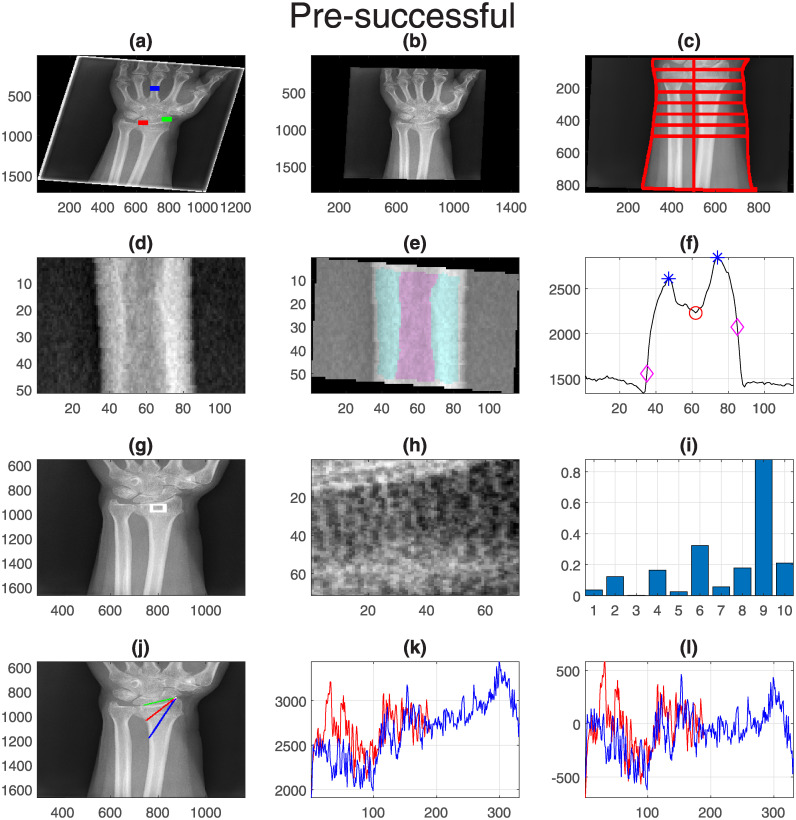
Pre-successful: One representative case with all its measurements. (a) Original with landmarks, (b) Rotated and collimator lines removed. (c) Boundaries of the forearm and width lines. (d) Central finger. (e) trabecular and cortical regions. (f) Intensity profile. (g) Textured region. (h) Detail of textured region. (i) LBP coefficients. (j) Location of intensity profiles. (k) Intensity profile values. (l) Adjusted intensity profiles.

**Fig 12:**
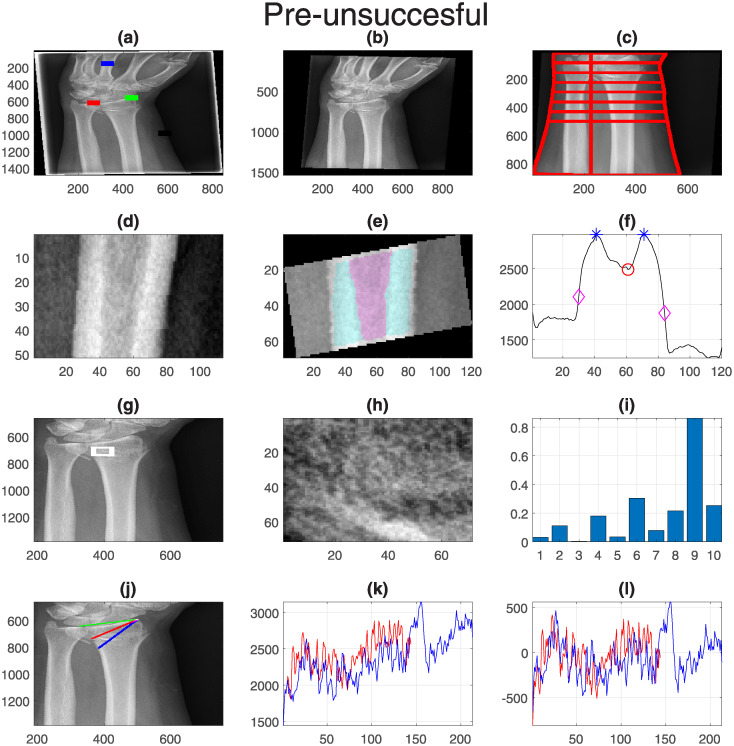
Pre-unsuccessful: One representative case with all its measurements. Refer to Fig 11 for the caption.

**Fig 13:**
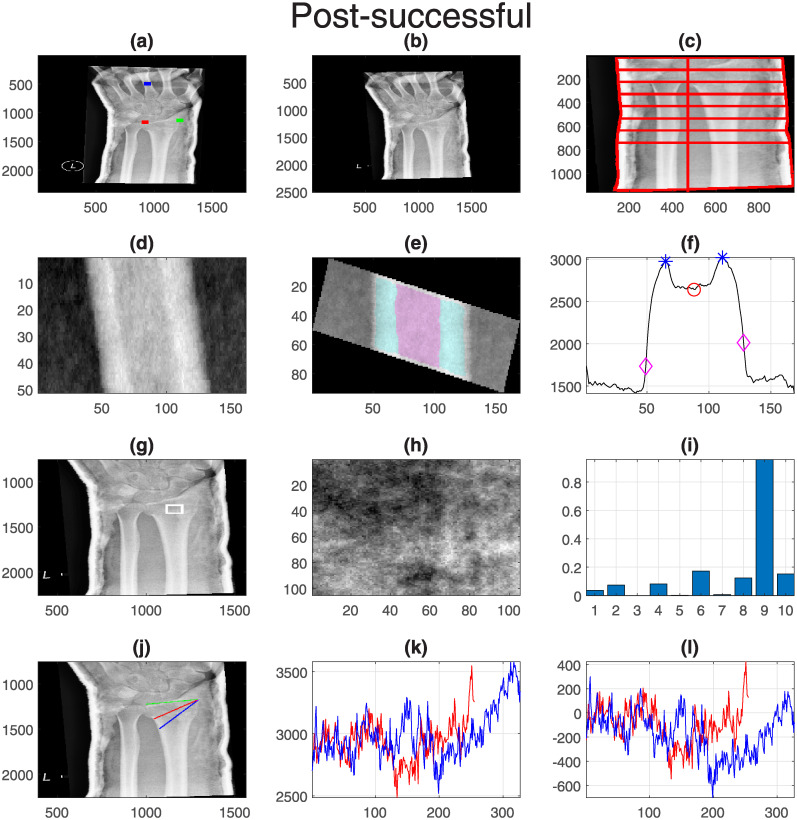
Post-successful: One representative case with all its measurements. Refer to Fig 11 for the caption.

**Fig 14:**
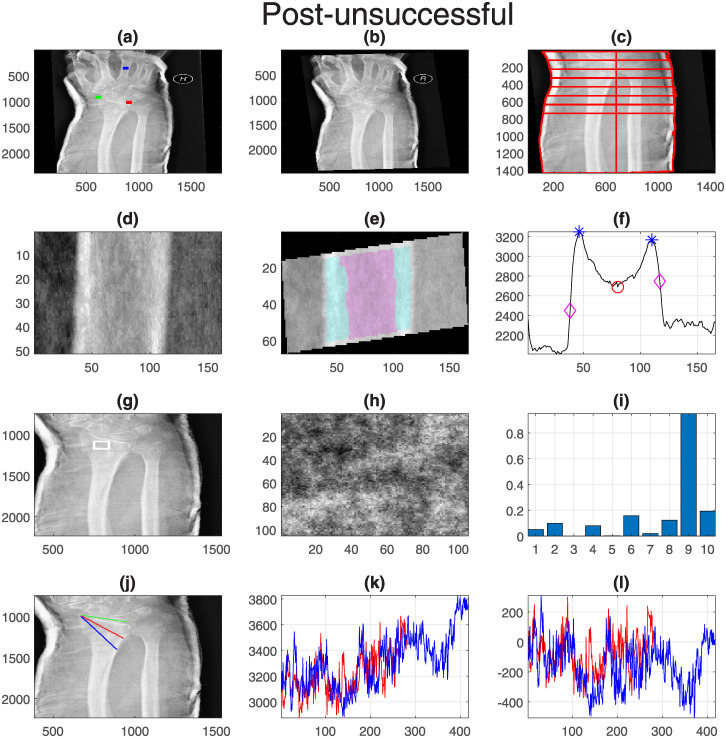
Post-unsuccessful: One representative case with all its measurements. Refer to Fig 11 for the caption.

**Fig 15:**
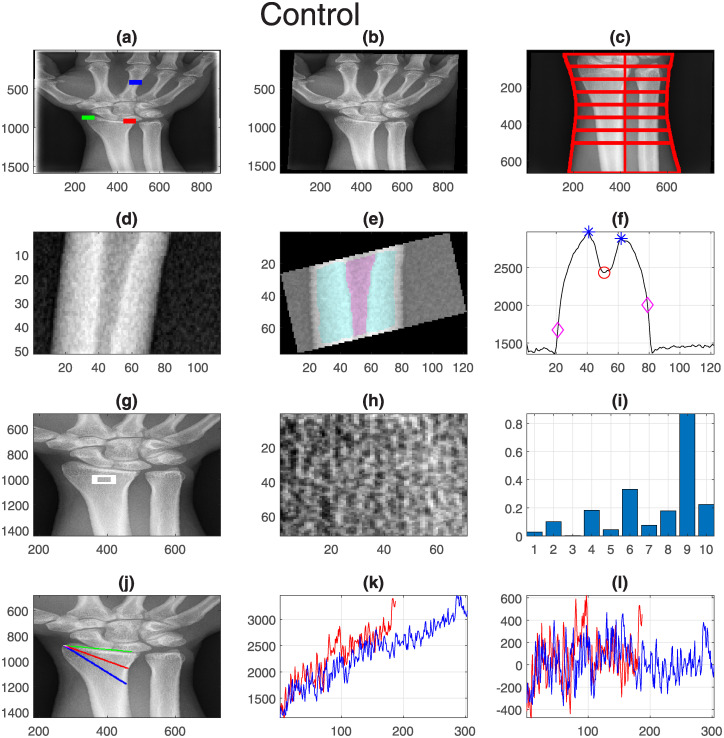
Control: One representative case with all its measurements. Refer to Fig 11 for the caption.

Finally, one representative case for each group was selected and displayed with all its measurements. These are shown in Fig 11 (Pre-successful), Fig 12 (Pre-unsuccessful), Fig 13 (Post-successful), Fig 14 (Post-unsuccessful) and Fig 15 (Control). Some observations between the measurements of the boxplots and the figures should be noted. The measurement of width (c) is greater than 1 when the extreme lines (1,8) are larger than the central ones (4,5). This is the case for the pre-successful and control examples, but less so for the rest. The slope of the short profile (i.e. the red line in (k)), is close to zero (i.e. horizontal) for both Post- cases, slightly higher for the Pre- cases but very high for the control case. Closely related is the standard deviation, which will be smaller for lines that are horizontal than for those that grow. Finally, the fourth coefficient of the LBP can be compared in (i). As in the boxplots, the Pre- cases and control are much higher (close to 0.2) than the Post- cases (close to 0.1).

## Discussion

In this work, a series of measurements were extracted from x-ray posterior-anterior images with a semi-automatic methodology. User intervention was minimal, and required the selection of three landmarks, which took less than one minute per image. All other steps were automatic and processing of each image took approximately 10-20 seconds. All measurements were validated visually. It should be highlighted that there was a considerable variation in the quality of the images. However, this did not affect the measurement extraction. Namely, labels such as those visible in Fig 6 and the presence of plaster casts did not affect the methodology.

No measurement indicated a statistical difference between the following groups: successful and unsuccessful, pre-intervention successful and pre-intervention unsuccessful, post-intervention successful and post-intervention unsuccessful. However, numerous measurements were statistically different between the groups: healthy controls and patients, pre-intervention and post-intervention for successful, unsuccessful and combined. The differences between healthy controls and patients could be expected due to many factors, such as the age of the patients being higher than the controls. Within the patient groups, the texture features, both those extracted from the profile lines as the LBP features, showed a statistical difference between controls and patients, as well as between x-rays of pre- and post- intervention. Twenty-five of the 32 measurements indicated statistical differences between controls and patients. Similarly, 21 measurements indicated differences between pre-intervention and post-intervention successful and 14 pre-intervention and post-intervention unsuccessful. The LBP measurements were most distinct showing differences for four of the seven groups, followed by the measurements derived from the intensity profiles. These results are encouraging and suggest that the texture features should be further studied, especially analysing the texture in different regions or larger areas as the differences could vary if the location was changed as has been reported in cases of bone mineral density [45] and following for longer periods as the changes in texture are not likely to be changes of osteoporosis given the short time between the pre- and post- imaging.

Whilst none of the results between successful and unsuccessful were significant, some to the texture measurements were close to 0.05. These results are also encouraging, and invite for further experimentation with larger samples and more measurements. New measurements such as radial shortening [46], volar and dorsal displacements [47], ulnar variance, palmar tilt and radial inclination [48] should be explored as these are widely used, but always extracted manually. Similarly, more osteoporosis-related measurements e.g. cortical thickness, internal diameter, cortical area [49] should be explored in regions other than the middle finger. Furthermore, several aspects should be considered in future research. The results can be further explored with statistical and machine learning techniques to determine the most discriminant features and consider new features to be extracted [50]. Also, the prospect of replacing the semi-automatic nature of the methodology with a fully automatic should be explored. This includes the possible option to incorporate the use of convolution neural networks for automatic detection of the radius [51, 52] and fracture diagnosis [53].
